# Study protocol for a randomized controlled trial of a group cognitive-behavioral course for depressed adolescents

**DOI:** 10.1186/s12888-016-0954-y

**Published:** 2016-07-18

**Authors:** Thormod Idsoe, Serap Keles

**Affiliations:** Norwegian Center for Child Behavioral Development, P.O. Box 7053, Majorstuen, 0306 Oslo Norway

**Keywords:** Depression, Adolescents, Group-CBT, Randomized controlled trial

## Abstract

**Background:**

School dropout is considered a serious problem in high schools in Norway. Despite studies which emphasize the importance of mental health as a unique risk factor for dropout, interventions have only taken this into account to a limited extent. Depression is one of the most prevalent mental health issues. Here we report the study protocol of a cluster randomized controlled trial of a group-CBT intervention, “*Adolescent Coping with Depression Course*” (ACDC) for depressed adolescents in upper secondary school.

**Method:**

The aim of this study is to investigate the extent to which ACDC can reduce depressive symptoms, prevent dropout and improve academic and social functioning among adolescents in upper secondary school. This study investigates the effectiveness of ACDC through a cluster randomized trial, in which course leaders are randomized to experimental or control conditions where the control groups receive usual care.

**Discussion:**

The intervention is expected to reduce depressive symptoms among adolescents. The study will further investigate whether the intervention can prevent dropout and improve academic and social functioning among adolescents in upper secondary school.

**Trial registration:**

ISRCTN registry ISRCTN19700389. Registered 6 October 2015.

**Electronic supplementary material:**

The online version of this article (doi:10.1186/s12888-016-0954-y) contains supplementary material, which is available to authorized users.

## Background

Dropout is considered a serious problem in upper secondary schools in Norway [1]. Despite international studies which emphasize the importance of mental health as a reason for school dropout [2, 3] Norwegian interventions have only taken this into account to a limited extent [4]. In general fewer than 20 % of adolescents who are in need of help for mental health problems have been in touch with health services for this reason [5]. On the basis of this, we assume that a relatively large group of students that we meet every day in our schools in Norway do not get proper support to cope with their school functioning. Depressive symptoms are among the most common forms of mental health problems in adolescents [6]. The overall aim of the proposed study is to investigate the effectiveness of a group-based cognitive behavioral therapy (CBT) intervention for depressed adolescents, *Adolescent Coping with Depression Course* (*ACDC*), in upper secondary school, and to see whether it has an impact on dropout rates.

### Dropout is strongly related to mental health

One third of Norwegian students drop out of upper secondary school [1] with the highest rates for vocational students [7] and immigrants [8]. The dropout rates are problematic because of the increasing need for formal education in order to get a job [4]. In Norway about one-third of those who are considered disabled on the basis of a mental illness have never received any treatment, and depression is the main reason for the disability [4, 9]. Studies suggest mental health as a unique risk factor for dropout [2, 3]. One study from the USA estimates that over half of those who drop out of secondary education have a diagnosable mental disorder [3]. In a study from one Norwegian upper secondary school, 20.8 % of the dropouts reported mental health problems as the cause [10]. In general, as many as 15–20 % of Norwegian adolescents report clinical range mental health problems [6], but only 16–17 % of those 15–20 % have consulted mental health services [5, 11]. This means that a large group of young people in need of help do not receive it, which again may cause additional challenges for schools in supporting this large group of “unhelped” students all the way from start through graduation. Finding better ways of supporting students at risk may also be beneficial for school motivation because of the relationship between academic issues and mental health [12, 13].

### Depression the most common mental health problem

According to the World Health Organization one of the most common disabling diagnoses is depression [14] with a peak age for first onset ranging from 15 to 21 years [14, 15]. In Norway this is one of the most common forms of mental health problem among children and young people [6], and the number of adolescents being disabled by depression is increasing [16]. Research findings from Norway also show more academic discrepancies and higher prevalence of mental health problems among immigrant youths, compared to their host peers throughout elementary, secondary and high school years [17]. Depression and depressive symptoms are characterized by impairments in cognitive, emotional and social functioning. Precisely these things are highly important for academic and social success. Targeting depression could thereby be highly relevant to decreasing dropout rates and increase academic motivation among students.

Depression is associated with a range of problems, like school dropout, school difficulties, health problems, increased substance abuse, suicide, as well as problems with peers and family [18–21]. Furthermore, it seems clear that the need for mental health care for young people is unmet [5]. Of more concern is that failure to seek early treatment is associated with longer disease course and more relapsing episodes [22]. Even subclinical depression among adolescents is associated with increased risk of depression and suicide attempts in adulthood [23]. In Norway, the mental health services and the municipalities have been strengthened in areas such as early identification of illness, prevention and early treatment [24]. This has been extended in recent government documents putting the focus on early intervention even higher. However, specialist mental health services still only seem to reach a minority of these adolescents [11]. Altogether, the prevalence estimates of depression as well as the public health consequences, high dropout rates and the failure to seek treatment suggest that effective interventions targeting adolescents with symptoms of depression need to be provided in places where they can be accessed – such as school.

### Schools as arenas for supportive interventions

A larger number of depressed students could probably be accessed if supportive interventions were provided closer to school. Schools are optimal places for such purposes as they provide access to all children, including those targeted for intervention, on a regular basis and over time. Immigrant students, who underuse mental health services [25] may gain particular advantage from school-based interventions. Group-based approaches could very well be implemented in relation to schools, either by the educational psychological service, school nurses or counselors/special educators at school. This could also counteract some of the unequal access to treatment related to social status. Investigations from Statistics Norway show that people with lower education receive little help from psychologists within the specialized services [26, 27].

### Group- based cognitive behavioural therapy (CBT) interventions

Some of the most effective interventions in treating depression among adolescents are based on cognitive behavioural therapy (CBT) [28]. Within CBT the main target is to change and modify maladaptive cognitions and behaviours that constitute the three core processes of depression [29]. Group-based CBT interventions for depressed adolescents have provided efficacy for adolescents with diagnosis of major depression or dysthymia [30–33] but also when used to prevent further development of subclinical depression among adolescents [34, 35].

## Methods

### Aim of the study

The aim of this study is to evaluate the *Adolescent Coping with Depression Course* (*ACDC*), a CBT program, compared to practice-as-usual control treatment in reducing depressive symptoms and preventing dropout among upper secondary school students, as well as increasing their academic and social functioning. Our main research questions are: 1) What are the effects of ACDC on depression, dropout, achievement and social functioning among students in junior high school? 2) Is immigrant status a possible moderator of the effects of ACDC?

### Design

**Fig. 1:**
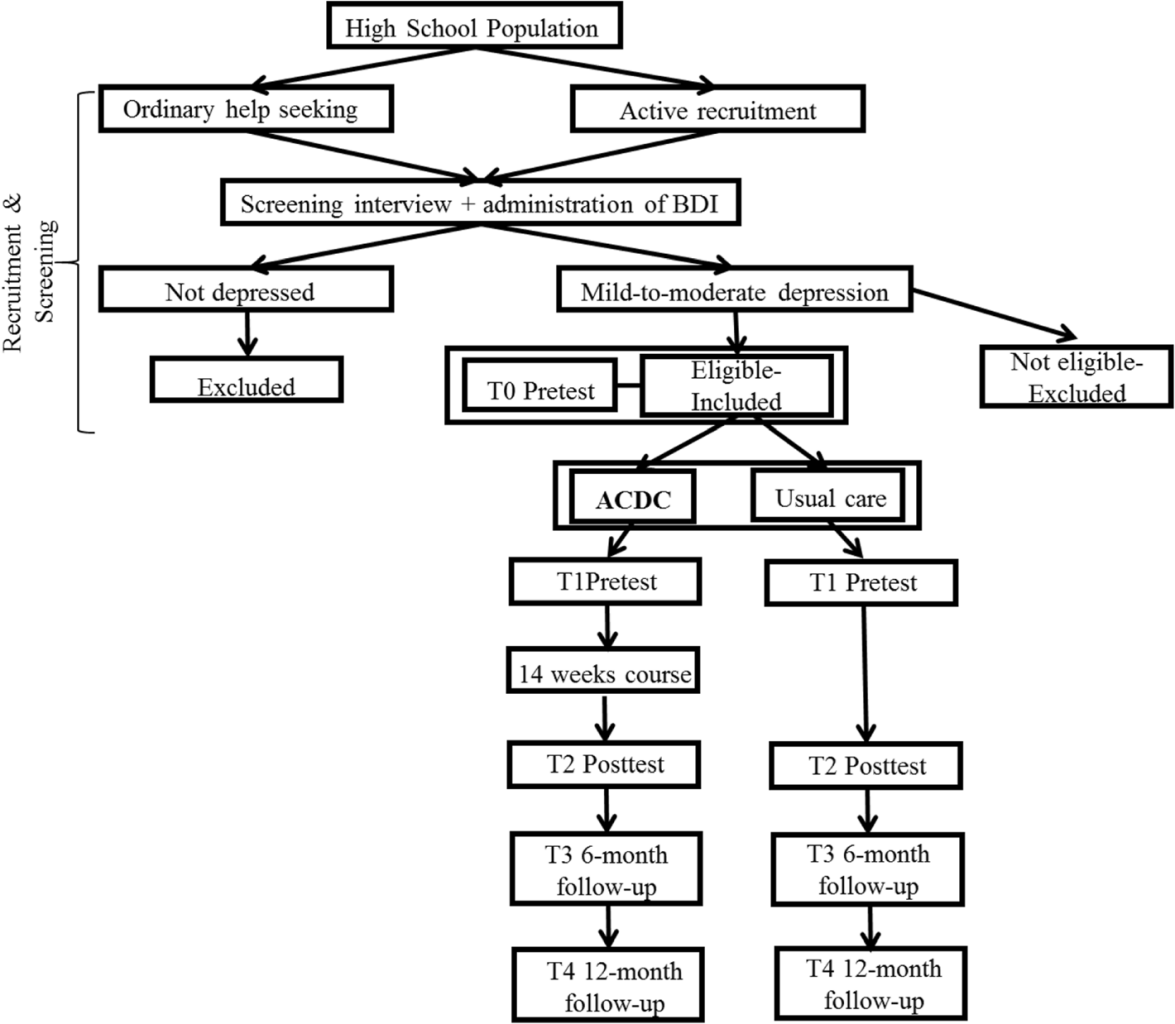
Overview of the study design

Figure 1 presents the general design of the study, and see also Table 1. Assessments are administered at initial screening (T0), before the ACDC starts (T1), after the course finishes (T2) and 6 months (T3) and 12 months (T4) follow-ups.

**Table 1:** World Health Organization Trial Registration Data Set

Data Category	Information
Primary registry and trial identifying number	ISRCTN registry ISRCTN19700389
Date of registration in primary registry	6 October 2015
Secondary identifying numbers	Protocol/serial number: 238081
Source(s) of monetary or material support	The Norwegian Research Council
Primary sponsor	The Norwegian Research Council
Secondary sponsor(s)	The Norwegian Center for Child Behavioral Development
Contact for public queries	Görel Bringedal g.e.bringedal@atferdssenteret.no
Contact for scientific queries	Thormod Idsoe thormod.idsoe@atferdssenteret.no
Public title	Effectiveness study of a CBT based adolescent coping with depression course
Scientific title	Effectiveness study of a CBT based adolescent coping with depression course to prevent dropout in upper secondary school
Countries of recruitment	Norway
Health condition(s) or problem(s) studied	Mild/moderate depression among adolescents
Intervention(s)	Active comparator: CBT-based group intervention, 8 consecutive weekly sessions of 120 min, two follow-up sessions conducted about 3 and 6 weeks after the last session, lasting approximately 90 min
	Placebo comparator: Usual care
Key inclusion and exclusion criteria	Cluster-randomized design where the course-leaders are recruited from the School Health Service, Public Health Nurses, the Educational and Psychological Counselling Services (PPT). They are then taught how to recruit a group of 1st or second year students from Upper Secondary School (Videregående skole). A complete assessment of possible participants (adolescents) has to be made by the course instructors before acceptance into the study.
	Inclusion criteria for adolescents:
	1. First or second year of upper secondary school (about 16–17 years old)
	2. Who have subclinical depression or mild to moderate major depressive disorder (MDD), according to the criteria of the DSM
	3. Normal intellectual functioning
	4. Normal reading abilities, and that was evaluated through the interview.
	Exclusion criteria for adolescents:
	1. Bipolar disorder
	2. Psychosis
	3. Substance-use
	4. ADHD or ADD
	5. Brain damage
	6. Danger of suicide
	7. Adolescents who are easily agitated
	8. Adolescents who lack the ability to function in a group
Study type	Single-centre cluster-randomized effectiveness trial with active control. Primary study design
	Interventional, Secondary study design
	Cluster randomised trial
Date of first enrolment	01/11/2015
Target sample size	35 clusters with 8–12 adolescents within each cluster
Recruitment status	Recruiting
Primary outcome(s)	1. Center for Epidemiological Studies Depression Scale, CES-D for adolescents, at Pre-test, T1, T2, T3, T4
	2. Dysfunctional Attitudes Scale (DAS) (short version) at Pre-test, T2, T3, T4
	3. Automatic Thoughts Questionnaire (ATQ) (short version) at Pre-test, T2, T3, T4
	4. Ruminative Response Scale (RRS) (short version) at Pre-test, T2, T3, T4
	5. Emotion regulation at Pre-test, T2, T3, T4
	6. Dropout (from school and official registries) at T1, T2, T3, T4
	7. Grades at T1, T2, T3, T4
Key secondary outcomes	1. Intentions to quit school at Pre-test, T2, T3, T4
	2. Social and Cognitive Competence at Pre-test, T2, T3, T4
	3. Life events at Pre-test, T2, T3, T4

Course leaders were recruited from the Educational Psychological Services, School mental health offices, and so on. To become certified as an ACDC course leader, one must have completed a minimum 3–year college/university education before attending the 5 day certification course. The trainers are either doctors, psychologists, nurses, educators, social workers, occupational therapists or physiotherapists. All are employed in community health, public health/public hospitals, schools or in private healthcare.

The course leaders have been randomized to experimental and control conditions by administrative personnel at the Norwegian Center for Child Behavioral Development. The control groups of adolescents will receive practice as usual, so those course leaders will be offered their training shortly after the experimental groups have finished after 14 weeks. Our design is thereby a cluster randomized trial where groups (course leaders) rather than individuals are randomized.

Participants (adolescents with depressive symptoms) are recruited by the ACDC trainers (both intervention and “active control” ones), and they are offering their own courses by placing information on schools and health centers, providing information through GPs, advertisements in local newspapers, on websites for youth, information on the local hospital’s medical meetings. Participants are recruited by either themselves take direct contact with course retainers, or they are referred by health visitors, GPs, psychiatric clinics, municipal services, school counselors, and the like. It will be established with doctor/referring in terms of differential diagnosis and considering further action after the course if necessary.

Fifteen course leaders for the intervention group received their training for a full week in October 2015, while the 15 leaders of “practice as usual” received a one-day training in how to recruit adolescents in October. Adolescents have been recruited throughout November and December 2015. Following a screening interview, baseline measures of all study measures was administered (see Table 2). The trial of the first cohort started in January 2016 and ran for 14 weeks, either by 10 sessions of the ACDC (treatment condition) or “practice as usual” for the control condition. Two weeks before the trial, the main outcome measure of depression was assessed. In addition to post-test in spring 2016, we will collect 6- and 12- month follow-ups. Personal number will be kept for five years in order to collect relevant information from public registers to further examine dropout.

**Table 2:** Measures to be administered at each measurement occasion

Measure/Scale	References	S	T0	T1	T2	T3	T4
Beck Depression Inventory	Beck et al. 1961 [42]	X					
Demographics			X				
Center for Epidemiologic Studies Depression Scale (CES-D)	Radloff, 1977 [44]		X	X	X	X	X
Dysfunctional Attitudes Scale (DAS)	De Graaf et al., 2009 [53]		X		X	X	X
Ruminative Response Scale (RRS)	Nolen-Hoeksema & Morrow, 1991; Treynor et al., 2003 [55, 56]		X		X	X	X
Automatic Thoughts Questionnaire (ATQ)	Hollon & Kendall, 1980; Netemeyer et al. 2002 [51, 52]		X		X	X	X
Emotion Regulation Questionnaire (ERQ)	Gross & John, 2003 [58]		X		X	X	X
Hopkins Symptom Checklist (SCL-10) anxiety items	Strand et al., 2009 [49]		X		X	X	X
Aggression	Roland & Idsøe, 2001 [50]		X		X	X	X
Intentions to quit school	Studsrød & Bru, 2009 [48]		X	X	X	X	X
Cognitive Competence	Harter, 1985; Paulsen et al., 2006 [46, 47]		X		X	X	X
Social Competence	Harter, 1985; Paulsen et al., 2006 [46, 47]		X		X	X	X
Social activities			X		X	X	X
Absence			X		X	X	X
Life events	Ystgaard, 1997; Murberg et al., 2004 [59, 60]		X	X	X	X	X
Satisfaction with the treatment Questionnaire	Ginsburg & Drake, 2002 [66]				X		

*S* screening, *T0* pretest Nov/Dec2015, *T1* Jan2016, *T2* June2016, *T3* Dec2016, *T4* June2017 Next cohort, *T0* Pretest Nov/Dec2016, *T1* Jan2017, *T2* June2017, *T3* Dec2017, *T4* June2018

### Participants and recruitment

Approximately 8–12 adolescents of both genders are considered a preferred group size, and 30 groups will be recruited. The target population is students from the 1^st^ or 2^nd^ grade from upper secondary school, who are 16/17 years old. There is a maximum cut-off age of 20 years and subjects must have subclinical depression or mild to moderate depression, according to the criteria of the DSM. Inclusion will be evaluated through a semi-structured interview by the course instructor/control leader. Generally, the participants will be recruited through their general practitioner, the School Health Service, public health nurses, the Educational and Psychological Counselling Services (PPT) or the Children and Young People’s Psychiatric Out-patient Services (BUP). Exclusion criteria include presence of bipolar disorder, psychosis, substance-use, ADHD or ADD and brain damage as listed in the ACDC manual.

### Intervention: The “Adolescent Coping with Depression Course”

The “Adolescent Coping with Depression Course” (ACDC) is a CBT-based group course for adolescents with subclinical or mild to moderate depression developed for the Norwegian context [36]. The development was funded by the Norwegian Directorate of Health (HDIR). In the first effectiveness study [37] the intervention has demonstrated effect sizes of medium to large reductions in symptoms of depression. The reduction was maintained or even increased at follow-up six months later. It would be interesting to see whether this intervention can prevent dropout among upper secondary school students. The intervention also needs to be tested within an RCT design.

ACDC is based on a new and updated understanding of depression within CBT, and the program contains different approaches and methods taken primarily from the work of Rational Emotive Behaviour Therapy (REBT) [38] and Cognitive Behavioural Therapy (CBT) [39]. However, ACDC also includes elements from Meta-Cognitive Theory (MCT) [40] and Positive Psychology (PP) [41] in addition to modern neurobiological perspectives. Within Meta-Cognitive Theory the focus is on how to modify the experience and regulation of your own thoughts [40]. You learn how to reflect on your own thinking style within a meta-perspective, by being consciously aware of your thinking and how you can think about your own thoughts. It is also important to be aware of how thoughts can change in terms of quality and style, and how to deal with this. Positive psychology is used for breaking thinking patterns, on the basis of a neurobiological understanding of how thinking patterns, behaviour and emotions are formed and developed. Another important component in the course is affect regulation. Further, the neurobiological perspective is used to give adolescents a better understanding of how information is processed and why we react the way we do, sometimes automatically. With these conceptual understandings, the adolescents can root their work with the concrete techniques in a solid base. There is a focus on symptom relief within a systemic perspective, meaning family-school-workplace, and separate pamphlets are distributed to those.

The importance of practising the techniques learned on the course is emphasized both in and outside the course setting to develop the necessary skills. One of the goals of the course is for the adolescents to acquire a ‘toolbox’ of skills and techniques to help them cope better with their depressive symptoms in the future. The material includes a manual for the facilitator, a course pamphlet for the participants, a pamphlet addressed to parents and also a pamphlet for the school/workplace as well. Additionally, a short downloadable presentation has been developed for teachers to use in class to present mental health as a theme if required. All the course materials are printed and published by the Norwegian Council for Mental Health (NCMH).

The course is usually delivered in a group format over 8 consecutive weekly sessions, each lasting approximately 120 min, with breaks. If necessary, it could be delivered twice a week over a shorter period. Two follow-up sessions are conducted about 3 and 6 weeks after the last session, lasting approximately 90 min. In total, the ACDC consists of 10 sessions. Ideally, the sessions are at the same time every week and each session has a specified topic described above. Generally, the sessions start by summarizing the previous session and reviewing the homework assignments.

### Control group: Practice-as-usual group

The participants in the control condition will not be offered any intervention, but they will receive treatment as usual. This will involve standard treatments, including no treatment or the use of pharmacotherapy, that the active control course leaders typically employ. The participants as well as the control leader will be asked about what they give/receive as usual care to examine the practice-as-usual in detail.

### Measures

Table 2 presents the measures that will be administered at each measurement occasion.

#### Screening measure

Potential study participants will be screened for eligibility and provided with study information in a semi-structured interview. At the first meeting with the course instructors/control leaders, adolescents will be screened using the Beck Depression Inventory (BDI; [42]) and will have a brief clinical interview. BDI is a widely-used self-report measure of depression and assesses a wide range of depression symptoms. It consists of 21 items with four levels of severity for each symptom. The BDI is reported to be a reliable and valid measure for depressive symptoms in adolescents [43]. Adolescents who have a BDI score ≥ 10, and who are between 16 and 20 years old can be included in the study. If they agree to participate, written consent will be obtained and the first pretest will be filled in by adolescents at the end of the interview.

#### Primary outcome measures

Dropout will be measured from school reports.

The level of depressive symptoms as assessed by the Center for Epidemiologic Studies Depression Scale for adolescents (CES-D; [44]) will also serve as one of the primary study outcomes. CES-D asks for the frequency of symptoms during the last week of depressed affect (7 items), lack of positive affect (4 items), somatic and retarded activity (7 items), and interpersonal problems (2 items). A four-point Likert scale is used (total score range: 0–60). A previous confirmatory factor analysis supported the use of four dimensions invariantly to gender and differing ethnic backgrounds in Norway [45].

#### Secondary outcome measures

A variety of secondary outcome measures will also be gathered. Social and academic functioning will be assessed by various measures. While social functioning measures include *social competence* and involvement in *social activities* with peers, academic functioning will be assessed using the measure of *cognitive competence*, and *grades* provided by the school. Perceived cognitive and social competence will be measured using slightly modified subscales from Harter’s [46] Self-perception Profile for Children [47]. In addition to *self*- *and school*-*related absence*, *intentions to quit school*, 4-item scale developed by Studsrød & Bru [48] will also be measured. *Social anxiety* will be assessed using the adapted version of the Hopkins Symptom Checklist (SCL-10) anxiety items [49]. *Reactive aggression* will be measured using an adapted version of Roland and Idsoe’s reactive aggression scale [50].

#### Mediators

The frequency of *negative thoughts* will be assessed using the single-factor, short-form version of the Automatic Thoughts Questionnaire (ATQ; [51, 52]). It has been suggested that this short version of ATQ shows high reliability and validity, without deteriorating content domain coverage of the construct [52]. The intensity of *dysfunctional attitudes* will be measured with the revised version of the Dysfunctional Attitude Scale (DAS; [53]) which is one of the most commonly used instruments as a mediator of outcome in CBT for depression [54]. Two-factor solution with 17 items, consisting of ‘dependency’ and ‘perfectionism/performance evaluation’, demonstrated good reliability and convergent construct validity [53]. Revised version of the Ruminative Responses Scale (RRS;[55, 56]), a self-report measure of *rumination* with 10 items, will be used to measure two aspects of rumination, reflecting and brooding. This version of RRS does not include items that seem to overlap with the measures of depressive symptoms [56]. *Emotion regulation strategies* will be assessed through the Norwegian version of the Emotion Regulation Questionnaire (ERQ; [57, 58]). The ERQ is a 10-item measure that assesses the use of two common emotion regulation strategies: cognitive reappraisal and expressive suppression.

#### Moderators

Research in Norway show higher prevalence of mental health problems among young people of immigrant origin compared to native peers throughout elementary, secondary and high school years [17]. Therefore, we will also explore whether *immigrant status* moderates the effect of the intervention. Adolescents will be asked to provide information about themselves, their parents and grandparents to evaluate their immigrant status and generation in detail.

The moderating role of *stressful life events* will also be assessed using the adapted version of the established life event lists for young people [59, 60]. The checklist consists of 10 pleasant or unpleasant events that an adolescent may have experienced in the past six months.

Course leaders will be asked about fidelity and attitudes, and how they provide the intervention retrospectively.

### Sample size and power calculations

Because the design is a single-centre cluster-randomized effectiveness trial with active control, where groups rather than individuals are randomized, possible cluster effects must be accounted for when calculating appropriate sample size [61]. We approximated cluster effects influencing our main outcome (depressive symptoms) by taking advantage of data from the existent study on ACDC [37], and calculated intraclass correlations (ICC’s) with the course groups as cluster. ICC for depressive symptoms was .08. Our calculations showed that with a .05 level of significance, power = .80, ICC = .08, number of clusters = 25, cluster size = 8, *N* = 200, we may be able to detect effect sizes of .49 (about medium effects according to (Cohen, 1988). We recruited 30 course leaders rather than 25 and we can withstand a total waiver of five of the groups while still identifying the effect size we have calculated.

### Data management

Cases are labelled with 11-digit social security codes throughout the project period. Personally identifiable information is required to connect self-reported data with information from school and inquiries about grades and dropout. Pre-intervention data will be collected by trainers for groups, while follow-up studies after 6 and 12 months are collected directly from the Norwegian Center for Child Behavioral Development through the posting of link to an electronic questionnaire. The study will make use of two separated databases for storing research data and contact information. All participants will receive an ID number. Collected data will be stored in a research database. The data will be stored in a safe manner, where only employees at the center authorized by the research director has access. Contact information for young people, such as the social security number, participant’s name, phone number, email and address will be kept in a separate database, unrelated to the research database. Our supplier Confirmit buys physical capacity and locality in RackspaceUK outside London. The data will then be stored on servers at RackspaceUK. Rackspace UK is a subsidiary of Rackspace Inc in the United States. Rackspace Inc is Safe Harbor compliant. This can be verified through https://safeharbor.export.gov/list.aspx. The electronic data collection happens via email project done by questionnaires emitted from the contact database. When the questionnaire is answered, the information is sent to the research database. The databases are protected by two factor authentication, where both have a personal password and unique passwords used every time you log in. If adverse events occur, the course leaders will report to the project leader. More details are described in the application to the ethical committee.

### Statistical analysis

SPSS will be used for 1) descriptive analyses for each time point and 2) repeated measures General linear models (GLM) for estimation of intervention effects. Structural equation modelling (SEM) will be used for latent growth curve models (LGC) to investigate individual variations in level and change across time [62–64]. The LGC can be used for estimation of intervention effects by comparing intervention and control group data through multiple-group LGC. These results can be compared with results from GLM. The LGC can also be expanded to Latent growth mixture models to explore whether there are sub-groups of individuals constituting specific clusters with change patterns deviating from the average change pattern estimated by use of traditional LGC [65]. Predictors can be added to the trajectories. Missing data will be handled by the FIML procedure in Mplus (if MAR).

### Dissemination

The Norwegian Center for Behavioural Development (Atferdssenteret) and the Norwegian Centre for Learning Environment and Behavioural Research in Education (Læringsmiljøsenteret) are both national competence centers keeping existing networks within the fields of education and mental health. The Centre for Child and Adolescent Mental Health, Eastern & Southern Norway (RBUP Oslo) is one of the leading research centers within its field. This provides lots of possibilities to spread knowledge from this project throughout the country. Results will be published in international referee-based scientific journals with high impact, national and Scandinavian vocational journals, newspaper chronics, seminars, conferences and our own internet pages. Several of the members of the project group teach at master and PhD levels within psychology and special education.

## Discussion

This manuscript describes the study protocol of a cluster randomized controlled trial of a group-CBT intervention, “*Adolescent Coping with Depression Course*” (ACDC) with the aim of reducing depressive symptoms, and possibly prevent dropout and improve academic and social functioning among adolescents in upper secondary school.

Dropout is considered a serious problem in upper secondary schools, and studies underscore the importance of mental health as a reason. This study may have implications for practitioners by providing them better tools to support students with their mental health problems. By so doing, dropout may be prevented as well.

A large group of young people in need of help do not receive it. It is expected that this study can contribute to more accessible interventions for this group of people so that more of them can receive the help that they need.

It is also expected that the conclusions that will emerge from this study can contribute in general to the evidence base of treatment options for adolescents with mild to moderate depression. Hopefully the findings can also add to the training of mental health professionals and special educators by providing them with better tools to approach these problems.

In conclusion, the intervention is expected to reduce depressive symptoms among adolescents. The study will further investigate whether the intervention can prevent dropout and improve academic and social functioning among adolescents in upper secondary school.

### Study status

The intervention started in January 2016. Out of a total of 30 recruited course-leaders, only 19 was established due to difficulties in the recruitment process, and some with low cluster size. In order to fulfill the requirements following our power-calculations, more adolescents need to be recruited. Another training of course leaders will take place in the autumn of 2016, so that an additional intervention period can be executed. Recruitment procedures will be revised. The planned additional intervention will take place in the spring of 2017.

For the intervention in the spring of 2016, post-test assessments are scheduled to be completed by the end of June 2016.

## Additional file

Additional file 1: SPIRIT 2013 Checklist: Recommended items to address in a clinical trial protocol and related documents. (DOC 121 kb)
